# Prodromal Functioning of Migraine Patients Relative to Their Interictal State - An Ecological Momentary Assessment Study

**DOI:** 10.1371/journal.pone.0072827

**Published:** 2013-08-16

**Authors:** Jan H. Houtveen, Marjolijn J. Sorbi

**Affiliations:** Department of Clinical and Health Psychology, Faculty of Social and Behavioral Sciences, Utrecht University, The Netherlands; University of Würzburg, Germany

## Abstract

Smartphones were used in an online Ecological Momentary Assessment (EMA) design to test prodromal functioning relative to the interictal state in migraine patients. Eighty-seven participants completed an electronic diary 4 times daily during 3-6 weeks to monitor their migraine attacks. Twice daily the diary additionally included 16 multi-answer questions covering physical symptoms (30 items), cognitive-affective functioning (25 items) and external factors (25 items). Eight clustered prodromal features were identified in the current study: sensory sensitivity, pain/stiffness, fatigue, cognitive functioning, positive affect, negative affect, effort spent and stressors encountered. Per feature, individual change scores with interictal control days - excluding 24-hour post-attack recovery - were computed for six 12-hour pre-attack time windows covering three prodromal days. Linear mixed model (fixed-effect) analysis established significant increases in sensory sensitivity, pain/stiffness and fatigue, and a tendency for increased negative affect, in the 12 hours prior to the attack. Positive affect and cognitive functioning were impaired both in the 25-36 hour and - more strongly - in the 12-hour time window before the attack. No effects were found for effort spent and stressors encountered. Exploratory (random effect) analysis revealed significant individual differences in the change scores in sensory sensitivity, pain/stiffness, fatigue and negative affect. It is concluded that the prodromal change in migraine - relative to interictal functioning - predominantly exists within the last 12 hours before attack onset. Individual diversity is large, however. Future research should zoom in to identify prodrome development within the 12 pre-attack hours as well as to isolate individual patterns.

## Introduction

Migraine produces attacks of severe headache, typically unilateral, of a pulsating quality and accompanied by nausea and/or vomiting, photophobia and phonophobia [1], which last 4-72 hours and markedly hamper routine daily activity [1]. Evidence shows that migraine is more than an isolated pain disorder, with abnormality extending far beyond the headache attack [2,3]. A prodromal stage with more subtle symptoms precedes the attack proper for hours to 2-3 days [3,4]. Migraine patients may not recognize these changes in functioning as part of the attack [5], but acknowledgement of the prodrome helps to predict attack onset [3,6,7]. Appraisal of prodromal features is highly relevant for attack prevention [6], and the rising interest in preventive treatment of migraine [8] coincides with growing research attention for the migraine prodrome. Ultimately this research seeks to identify prodromal markers of an impending migraine headache, and the current study aims to contribute to this search.

It is well known that menstruation can trigger a migraine attack [1,9,10], but other prodromal features have also been identified. These include physical symptoms such as light and noise sensitivity, stiffness or pain in the neck (and shoulders or parts of the head not specific to migraine headache), fatigue or sleep disturbance, concentration problems and negative mood; other features include nutritional factors, weather conditions and perceived external demands or stressors [11–19].

Previous research on prodromal features in migraine suffers from two limitations, however. First, the majority of studies was based on retrospective report. This is a serious drawback, because the results of retrospective questionnaires are obscured by recall bias, selective memory and the need for causal explanations [11,20,21]. Instead prospective diary methods - preferably catching the momentary experience in daily life - are required to adequately identify the migraine prodrome. The appropriate methodology for this is Ecological Momentary Assessment (EMA), an innovative technique for real-time and just-in-time measurements of fluctuating states. In EMA, randomly generated alarms are used to prompt momentary responses several times per day [20], and this seems suitable to properly detect the precursors of the migraine headache. Second, most previous studies lacked individual baseline levels. To be counted as part of the migraine prodrome requires securing that a feature under study does not occur regularly. A prodromal feature thus emerges by its deviation from the state in the so-called interictal phase between attacks. This calls for longitudinal measurement, and for careful selection of control days per patient, in order to establish pre-attack change relative to individual baseline. EMA allows the assessment of both pre-attack and control days.

The following three studies employed prospective diaries to identify prodromal features of a migraine attack. Wöber et al. (2007) used a comprehensive paper-pencil diary for three consecutive months to explore a wide spectrum of attack precursors [22]. Every evening, 327 migraine patients filled in the diary irrespective of the presence of headache. The diary contained 52 items covering physical, psychological, nutritional and environmental factors. Results showed that a migraine attack was preceded in the day before onset by physical symptoms (muscle tension in the neck and fatigue) and psychic tension, and attacks were related to menstruation and several external factors such as atmospheric pressure or holidays. The employment of a paper-pencil diary limits these results, however, due to uncertain compliance and the absence of response-time information [21]. These shortcomings can be solved with electronic EMA data collection using handheld computers or smartphones.

Giffin et al. (2003) were the first to use electronic diaries programmed in handheld computers to study prodromal symptoms predicting an attack [7]. The study enrolled 120 participants with migraine (with 76 completers) during three months. Randomly once a day, the diary device generated an alarm to initiate assessment of momentary symptoms; in addition, participants could make voluntary entries when they experienced symptoms. A symptom was considered to be a correct predictor if it made the patient to expect an attack, and the headache indeed appeared within 72 hours. For each symptom, the percentage of ‘correct’ predictions was reported. The study identified light and noise sensitivity, stiffness of the neck, cognitive slowing and fatigue in particular as the strongest predictive symptoms of the migraine attack. This elegant study had certain limitations as well: the set of potential attack predictors was relatively restricted and did not (properly) cover affective functioning and stressors or other forms of external demand, comparison relative to individual symptom baseline level was not included, and the time window of 72 hours was rather global.

In the study of Hashizume et al. (2008) 16 patients with migraine kept an electronic diary four times a day for two weeks [23]. Fourteen of them experienced an attack in this period resulting in 27 attacks. Anxiety, depression and other self-reported affective states and stressors were compared between the three pre-migraine days and control days. Results indicated that the levels of self-reported affect and stressors did not differ between pre-migraine and control days. The limitations include the small number of participants and attacks as well as a relatively narrow range of potential attack precursors under study, the possibility that control days may have been inflated by the inclusion of recovery days after an attack, and the fact that specific time windows within the three days (72 hours) before attack onset were not distinguished.

The present study supplements the prior prospective investigations using smartphones with wireless internet, a comprehensive diary of the migraine prodrome and attack, and EMA software permitting control over response time and compliance. Two distinct advantages solve other limitations of earlier studies. First, six 12-hour time windows are distinguished within the three prodromal days to pinpoint symptom occurrence more closely. This also sheds light on the potential (progressive) course of attack precursors. Second, prodromal features are determined relative to their regular occurrence between attacks. For each 12-hour window, differences with matched interictal control days are computed per subject and per feature, excluding a 24-hour post-migraine recovery period. Valid precursors are indicated by deviations from matched control days based on these within-subject difference scores.

## Materials and Methods

### Participants

Participants were patients, aged between 18 and 70 years old, with migraine with or without aura according to the International Classification of Headache Disorders (ICHD-II) [1] assessed by standard questionnaire and a 4-week headache diagnostic diary. Inclusion criteria were: 1–8 attacks during 4 weeks (halfway the study this was confined to 2-8), no medication overuse, and absence of psychopathology according to clinical norms (SCL-90R total score <178). Exclusion criteria were: headache occurring on ≥15 days per month, migraine duration <1 year, and migraine onset at age >50 potentially referring to underlying organic disease. Participants were recruited through the Society of Dutch Headache Centers, the Dutch Society of Headache Patients and newspapers. Participants were allowed to use acute and prophylactic medication.

Ninety-three subjects participated. Six participants were excluded because they did not experience a migraine attack during the 3-week assessment period. Table 1 provides the demographics of the 87 participants who had reported at least one attack. The Medical Ethical Committee at Utrecht University Medical Center approved the electronic diary study, and all participants provided written informed consent.

**Table 1:** Participants’ demographics.

	(n=87)
Female/male ratio	74/13
Age (mean, range)	44.5 [25-68]
Education^#^	4.8 [3-6]
Migraine frequency / 4 weeks (mean, range)	3.5 [1-8]
Years of migraine (mean, range)	21.2 [3-58]
Females reporting menses within registration	n=43

Notes: Demographics are missing for one female subject; According to self-report 36.7% was familiar with migraine with aura but this was not formerly diagnosed; the groups without (N=54) and with self-reported aura (N=32) did not differ at all regarding these demographics;

^#^ mean on interval scale from 1 = elementary school till 6 = university master degree.

### General procedure and materials

The present study employs real-time electronic diary recordings obtained with a broader EMA software-application called online digital assistance (ODA). User-friendliness, acceptance and utility have been well established [24,25], and ODA consists of two components: real-time assessments of functioning including migraine prodromal features and attacks (online EMA), and direct support of migraine self-management in real life (online coaching) [24]. ODA was employed during three consecutive weeks as part of two trials testing the efficacy of a behavioral training (BT) in migraine self-management. In trial 1 BT was provided in a small-group setting by patient trainers [26,27]; in trial 2 BT was offered individually through the internet [28]. The ODA procedures and EMA measures were identical in both trials, and the 87 participants were equally distributed (trial 1: N=44; trial 2: N=43). The moment of ODA provision differed, however: it was offered in, respectively, the last three of 10 training weeks (trial 1) or three months after training completion halfway the 6-months follow-up period (trial 2). In addition, trial 1 offered ODA again at 6-months follow-up [25]. Unfortunately, we lost online EMA data after conclusion of trial 1 due to a fatal backup problem. Therefore the present study includes a limited part of the follow-up data of trial 1 obtained from 15 participants (12 female, 3 male, mean age = 44.4, with ≥1 migraine attack in the two assessment periods).

Diary keeping was conducted on a smartphone (PalmOne Treo 600^TM^ or Palm Treo 500^TM^, Palm Inc., Sunnyvale, CA, and Nokia C3^TM^, Nokia Corporation, Espoo, Finland). All diary entries were time-stamped, and a wireless UMTS Internet connection handled the transfer of questions and answers to a shielded server for data storage.

Participants were visited at home for a practice session, instructions and handing over of the smartphone. This visit initiated three weeks of EMA diary keeping four times per day. Twice daily the smartphone prompted the diary entry by means of a random alarm generated between 9:30-12:00 in the morning, and between 13:30-16:00 in the afternoon. In case of non-compliance the alarm was repeated after 5 and 10 minutes. In addition, the participants kept an early morning diary after getting up, and an evening diary at bedtime, at their own moment of convenience. All four diaries per day assessed the presence of migraine. The two alarm-controlled diaries additionally assessed prodromal features; the early-morning and evening diaries additionally covered a brief review of the past night or day. At the end of the EMA period, participants were visited at home again for a debriefing session and return of the smartphone.

### Registration of migraine attacks

The presence of a migraine attack was established four times daily according to the ICHD-II classification criteria [1]. The diary assessed the occurrence of an aura and of headache, and for headache established the intensity (mild/moderate/severe), quality (throbbing or pulsating versus tightening, pressing or stabbing) location and laterality of the pain, impact of routine physical activity, and accompanying symptoms (nausea, vomiting, photo- and phonophobia). Attacks were supposed to have started with the first migraine diary and to have stopped with the first migraine-free diary, which in turn initiated a 24-hour recovery period.

### Registration of prodromal features

The migraine prodrome was assessed in the morning and afternoon by means of 16 multi-answer questions covering 80 potential characteristics (physical: N=30; cognitive-affective: N=25; external [demands, weather, nutritional]: N=25). Questions focused on momentary states and were introduced with “right now I feel/have/experience…”. Questions for current activities focused on the moment of the alarm (for which the colloquial term ‘beep’ was used), and questions concerning nutritional factors and external demands captured the episode since the last alarm. Answers were provided by ticking off appropriate alternatives, which was converted in dichotomous [0/1] scores. These scores were combined based on a PCA factor analysis (selected eigenvalue >1; oblique rotation) across all available observations (centered for subject means). This yielded eight clustered premonitory features with 4-7 items listed below according to factor loading.

#### Sensory sensitivity (6 items)

Right now I experience - difficulty reading | - blurred vision | - sound sensitivity | - dizziness | - smell sensitivity | - a sensitive skin.

#### Pain/stiffness (4 items)

Right now I have - pain in my forehead a/o back of my head | - stiff or painful shoulders | - pain in my neck | - a stiff neck.

#### Fatigue (7 items)

Right now I have strained eyes. Right now I feel - weary | - rickety | - tired | - exhausted | - lifeless | - sleepy.

#### Cognitive functioning (6 items)

Right now - I have things in order | - my head is clear. Right now I feel - alert | - well concentrated **|** - competent. At the moment of the beep I worked efficiently.

#### Positive affect (7 items)

Right now I feel - well | - cheerful | - inspired | - strong | - relaxed | - contented. At the moment of the beep I did something with pleasure.

#### Negative affect (7 items)

Right now I feel - tense | - dreary | - annoyed | - worried | - sad | - lonely | - angry.

#### Effort spent (6 items)

At the moment of the beep - I was working hard | - I felt strained (not at ease) | - I was busy | - much was expected | - I was exerting myself very much | - I was thinking hard.

#### Stressors encountered (4 items)

Since the last beep - something unpleasant happened | - I had a conflict | - I had a problem I couldn’t solve | - things went not my way.

### Data preparation and statistical analysis

Mean clustered feature scores were computed for each morning and afternoon diary entry, which yielded eight variables with a score ranging from 0.0 to 1.0.

Next, the time stamps for the start and end of the migraine attacks were determined in order to separate prodromal and control diaries. Counting backwards per attack, the time intervals (in hours) were computed for each diary entry within the 72 hours preceding the start of an attack. The diary entries in this time-window were considered prodromal diary entries. Not all prodromal diary entries were valid, however. Diary entries were discarded as invalid for three reasons. First, the three-day pre-attack windows of two attacks could overlap; in that case, pre-attack diary entries at issue were assigned to the earliest attack and were counted as invalid for the next attack. Second, invalid were pre-attack diary entries that extended into the 24-hour recovery period of a former attack. Third, missing entries directly preceding a migraine rating were considered invalid since it was unclear whether this concerned a prodromal diary entry or a migraine diary entry not recorded due to the severity of the attack.

All diaries entered more than 72 hours before or more than 24 hours after an attack were considered interictal control diary entries. Of these 93% were headache-free and 7% contained predominantly mild headache not classified as a migraine attack. The control diary entries were mean-aggregated per subject for the morning and afternoon entries and for weekend (Saturday and Sunday) versus week (Monday-Friday). This yielded four mean-aggregated control diary entries per subject. The valid prodromal diary entries were matched with the mean-aggregated control diary entries for subject, morning versus afternoon, and weekend versus week. Next, subject-specific differences (delta scores ranging from -1.0 to 1.0) were computed per variable. These delta scores were assigned to the appropriate 12-hour prodromal time-window (of respectively 0-12, 13-24, 25-36, 37-48, 49-60 and 61-72 hours before the attack). Per subject this resulted in pre-attack delta scores per time window for each clustered prodromal feature.

The data were analyzed using linear mixed model multilevel analysis with ML estimation (IBM SPSS v 20.0.0). First, the overall deviation from zero, as well as differences between the six delta scores, were tested across subjects (the fixed effects of an intercept and prodromal time-window). Mixed model multilevel analysis was needed to adequately take care of missing values and unbalanced data. The model included day (1–7), day-square, time point per day (0-24 in hours), time point per day square, and - feature-specific - mean control diary baseline values as covariates. Estimated marginal means of the non-transformed delta values were used for the graphs^10^; log transformed delta values were used for the statistical tests. Secondly, the extent of individual differences in the occurrence of the delta scores in the eight prodromal features were explored by testing specific subgroups and by means of time-contrast models with random slopes. The alpha-level was set at p<.05.

## Results

### Observed migraine attacks and valid diaries

#### General

**Figure 1:**
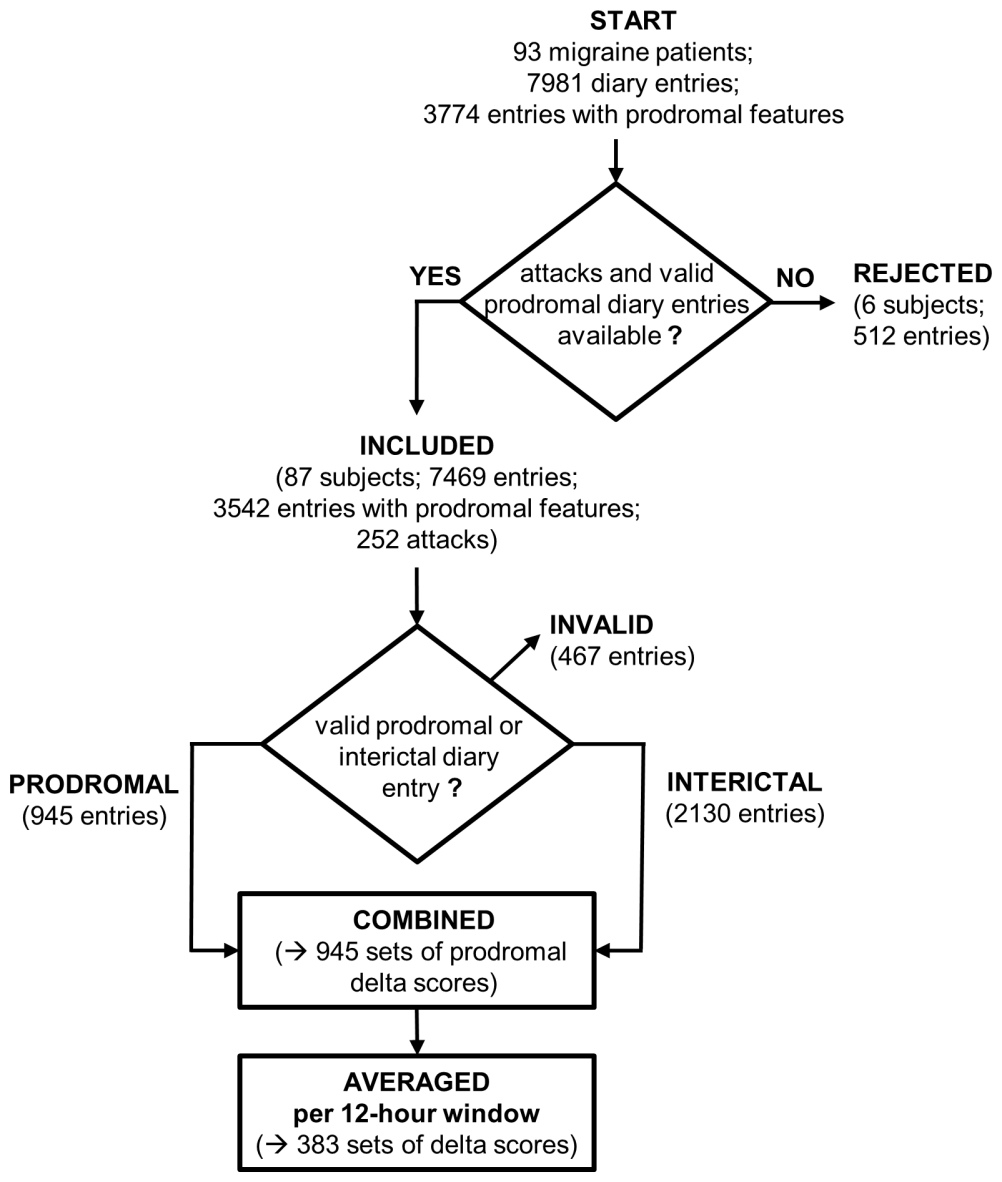
Flow chart for the selection of subjects and diary entries.

See Figure 1 for a flow chart. The 87 included participants produced 7469 valid time points with diary entries kept with an overall compliance of 89.5%. Compliance was equally distributed over the days of the week (χ^2^(6)=6.2, *p*=.40) but differed for the four time-points per day (χ^2^(3)=20.2, *p*<.001): it was highest in the early morning (n=1980) and late evening (1947) and somewhat lower in the morning (n=1779) and afternoon (n=1763).

#### Migraine attacks

The diary recording yielded 252 attacks with ≥1 valid corresponding prodromal diary entry (mean per subject: 2.9, range 1-9). Attack occurrence was not equally distributed over the four time-points per day (χ^2^(3)=11.7, *p*<.01), and significance remained after correcting for the differences in compliance (χ^2^(3)=8.7, *p*<.05): most attacks (78) occurred in the late evening, while this were 68 and 65 attacks, respectively, after getting up and in the morning, and least attacks (41) were reported in the afternoon. Attack occurrence also was not equally distributed over the week (χ^2^(6)=26.1, *p*<.001), and significance remained after correction for difference in compliance (χ^2^(6)=24.1, *p*<.001): most attacks started on Tuesday (46), Wednesday (49) and Thursday (48); occurrence was average in the weekend (Saturday: 34, Sunday: 35), while least attacks started on Monday (18) and Friday (22). Use of acute headache medication was indicated in 59.5% of the diary entries in which an attack was reported.

#### Valid prodromal and interictal control diary entries

This study contained 945 valid morning and afternoon prodromal diary entries (mean per subject: 11.0, range 3-27) that were equally distributed over the morning and afternoon without (χ^2^(1)=0.24, *p*=.63) and with correction for difference in compliance (χ^2^(1)=0.34, *p*=.56). Pre-attack diary entries - as were the attacks - were not equally distributed over the week without (χ^2^(6)=61.2, *p*<.001) and with correction for differences in compliance (χ^2^(6)=57.3, *p*<.001): most valid pre-attack diary entries were found on Mondays (184) and Tuesday (191); Sunday (112), Wednesday (131) and Thursday (119) were average, and the least valid diary entries were obtained on Friday (106) and Saturday (102). In total 2130 valid interictal control diary entries were retrieved that were equally distributed over the morning and afternoon (χ^2^(1)=0.0, *p*=.96). The mean number of control diary entries per day was 304 (range 293-334), and they were equally distributed over the days of the week (χ^2^(6)=4.3, *p*=.64). After subject-specific mean aggregation of interictal control diary entries matched with the prodromal diary entries regarding occurrence in, respectively, morning versus afternoon and week versus weekend day, 13 (3.7%) weekend control diary entries were missing and were substituted by matched individual week control diary entries. Acute headache medication was reported in 4.9% of the valid prodromal diary entries and in 4.1% of the interictal control diary entries.

#### Difference scores (delta)

Delta scores, representing the difference between prodromal diary entries and mean interictal control diary entries, were available for 383 prodromal 12-hour observations (mean per subject: 4.45, range 2-6). There were no significant differences in the distribution of the delta scores over the six 12-hour time windows (χ^2^(5)=0.89, *p*=.97).

### Results across subjects (fixed effects)

**Figure 2:**
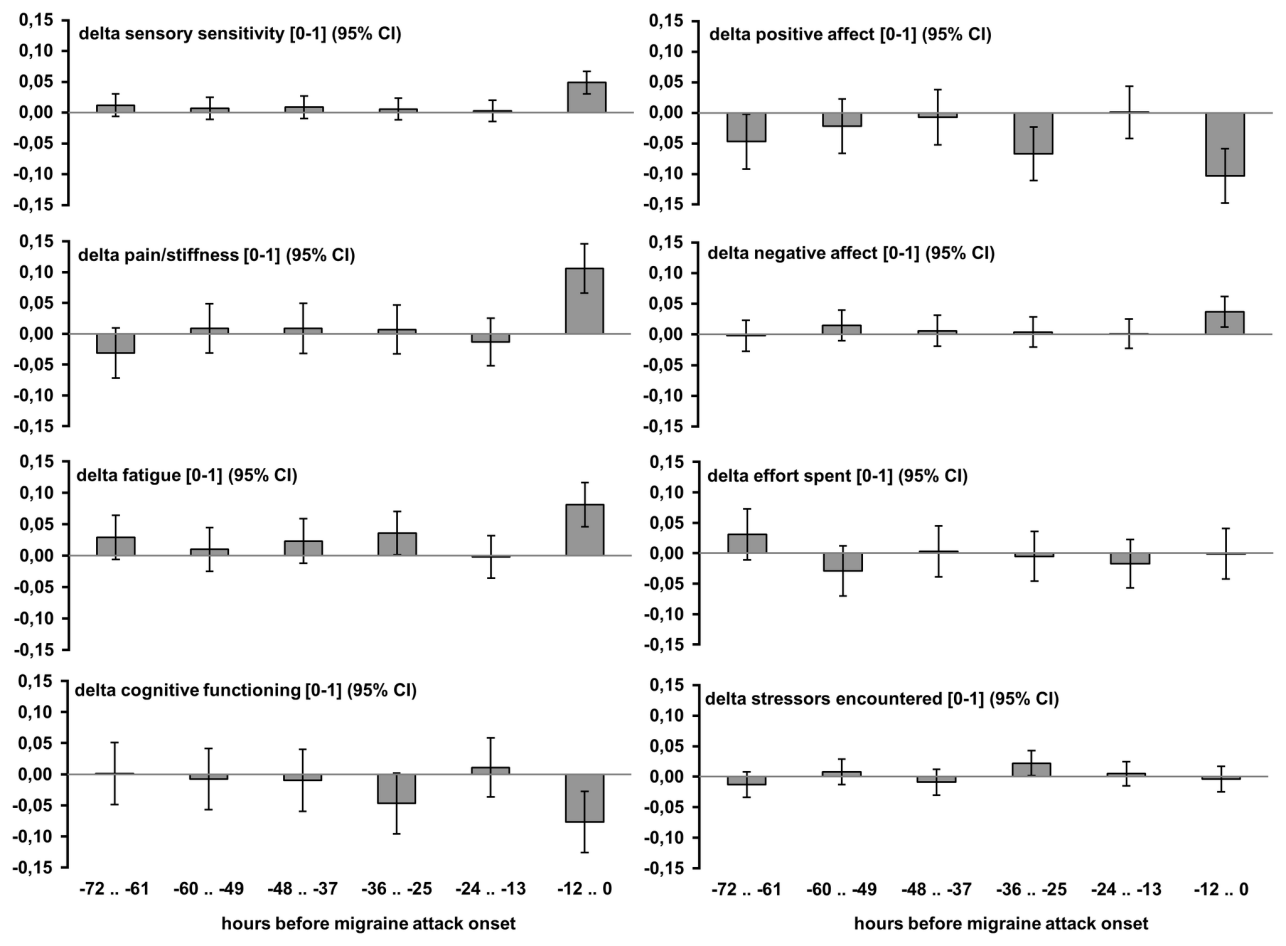
Estimated marginal means for the (aggregated) delta scores. Note: Day of the week, day-square, time, time square, and control day baseline values were included as covariates in the model.

The six 12-hour delta scores and the corresponding 95% confidence intervals are depicted in Figure 2. The intra-class correlation coefficients (ICC), indicative of the ratio of inter- versus intra-individual variances of the delta scores, are shown in Table 2. The relatively low ICC coefficients indicate that - due to the employment of delta scores - the variances were largely caused by intra-individual variation (at the residual time level). Linear mixed model analyses were performed for each of the eight clustered premonitory features to test for the significances of the (fixed factor) intercept and the difference between the six prodromal windows:

**Table 2:** Percentages of the residual (time level) variances explained by the model.

		FIXED effects		RANDOM effects	
	ICC	contrast	variance	contrast	variance
Δ sensory sensitivity	.05	000001	4.96^***^	000001	28.93^***^
Δ pain/stiffness	.19	000001	9.74^***^	000001	30.41^**^
Δ fatigue	.21	000001	3.96^***^	000001	14.77^*^
Δ cognitive functioning	.25	000101	3.42^**^	none	n.s.
Δ positive affect	.20	000101	3.55^***^	none	n.s.
Δ negative affect	.01	000001	2.42^*^	000001	18.04^**^
Δ effort spent	.23	none	n.s.	none	n.s.
Δ stressors encountered	.01	none	n.s.	none	n.s.

Notes: Five different time contrasts were explored: linear, 000001, 000011, 000101, and 000111 (see text); Results of the contrast with the best significant fit (largest explained variance on the time level) were reported; ICC = intra-class correlation coefficient; n.s. = not significant;

^*^ p<.05;

^**^ p<.01;

^***^ p<.001 (Wald Z test).

#### Sensory sensitivity

No significant effect was found for the intercept (*F*(1,382.2)=.01, *p*=.91), but a significant difference between the six prodromal time windows was found (*F*(5,333.9)=4.01, *p*<.01). Post-hoc tests showed that sensory sensitivity was increased in the 0-12 hour prodromal time window. The amount of variance explained (at the time level) was 5.2%. The covariate effects were all non-significant except for baseline sensory sensitivity (*F*(1,149.5)=5.48, *p*<.05).

#### Pain/stiffness

Again no significant effect was found for the intercept (*F*(1,358.1)=2.13, *p*=.15) but a significant difference was found between the six prodromal time windows (*F*(5,314.8)=6.88, *p*<.001). Post-hoc tests showed that pain/stiffness was increased in the 0-12 hour prodromal time window. The amount of variance explained (at the time level) was 10.9%. The covariate effects were only significant for baseline pain/stiffness (*F*(1,129.1)=25.57, *p*<.001).

#### Fatigue

Again, no significant effect for the intercept (*F*(1,359.9)=.36, *p*=.55) but a significant difference between the six prodromal time windows was found (*F*(5,316.7)=3.37, *p*<.01). Post-hoc tests showed that fatigue was increased in the 0-12 hour prodromal time window. The amount of variance explained (at the time level) was 5.7%. Except for baseline fatigue (*F*(1,123.5)=26.17, *p*<.001) covariate effects were non-significant.

#### Cognitive functioning

Again, no significant effect for the intercept (*F*(1,358.9)=.68, *p*=.41) but a significant difference between the six prodromal time windows was found (*F*(5,319.6)=2.60, *p*<.05). Post-hoc tests showed that cognitive functioning was decreased in the 0-12 and the 25-36 hour prodromal time windows. The amount of variance explained (at the time level) was 4.3%. The covariate effects were significant for baseline cognitive functioning (*F*(1,128.8)=43.90, *p*<.001).

#### Positive affect

No significant effect for the intercept (*F*(1,364.3)=.10, *p*=.75), but again a significant difference between the six prodromal time windows was found (*F*(5,324.5)=3.65, *p*<.01). Post-hoc tests showed that positive affect was decreased in the 0-12 and the 25-36 hour prodromal time windows. The amount of variance explained (at the time level) was 5.5%. The covariate effects were significant for day (*F*(1,382.5)=4.72, *p*<.05) day-square (*F*(1,381.8)=5.15, *p*<.05) and baseline positive affect (*F*(1,128.8)=72.60, *p*<.001).

#### Negative affect

No significant effect for the intercept (*F*(1,383.0)=.04, *p*=.84), and no significant difference between the six prodromal time windows (*F*(5,336.0)=1.49, *p*=.19) was found for negative affect. Post-hoc tests, however, showed that negative affect was increased in the 0-12 hour prodromal time window. This effect - with only 2.8% variance explained at the time level - did not reach significance in the overall test. The covariate effects were only significant for baseline negative affect (*F*(1,75.5)=52.36, *p*<.001).

#### Effort spent

No significant effect was found for the intercept (*F*(1,361.0)=.07, *p*=.79) and no significant difference was found between the six prodromal time windows (*F*(5,321.1)=1.35, *p*=.24). Effort spent was not different in the 72 prodromal hours relative to interictal baseline. The covariate effects were significant for day (*F*(1,381.4)=9.69, *p*<.01), day-square (*F*(1,381.0)=9.90, *p*<.01) and baseline effort (*F*(1,242.3)=65.24, *p*<.001).

#### Stressors encountered

Again no significant effect for the intercept (*F*(1,383.0)=1.26, *p*=.26) and no significant difference was found between the six prodromal time windows (*F*(5,336.5)=1.57, *p*=.17). In the 72 prodromal hours stressors encountered was not different from interictal baseline. The covariate effects were significant for baseline stressors (*F*(1,170.6)=92.55, *p*<.001).

### Exploratory analyses

The number of attacks (Spearman’s rho=0.04, *p*=.72) and the number of valid diary entries within the 72 hours preceding an attack (Spearman’s rho=0.19, *p*=.09) were not significantly correlated with age. Testing for group differences regarding, respectively, migraine with/without aura, reported menses (y/n), age (below/above 44 years median or with three age groups) and attack frequency (below/above 3 attacks per month median) yielded no significant (fixed effect) interaction effects of group and the six prodromal time windows for any of the prodromal features (all *p*’s >.05). Because only 13 males were included, gender differences could not reliably be explored.

Next, five different time contrasts were explored. First, a linear increase over time (from 72 hours before the attack till the start of the attack) was tested (i.e. linear). Second, a contrast was tested that was zero for each prodromal window except for the 0-12 hour time window just before the attack (000001). Third, a contrast was tested that was zero for each prodromal window excepting the 13-24 and 0-12 hour pre-attack time windows (000011). Fourth, a contrast was tested that was zero for each prodromal window excepting the 25-36 and 0-12 hour pre-attack time windows (000101). Fifth, a contrast was tested that was zero for the first three prodromal windows (000111). Table 2 presents the contrast with the best significant fit representing the largest percentage of explained variance. The largest amount of fixed factor variance was explained by an increase in the 0-12 hour prodromal time window for sensory sensitivity, pain/stiffness, fatigue and negative affect. Allowing a random slope added a substantial amount of the variance explained for these four features. For cognitive functioning and positive affect, the largest amount of fixed factor variance was explained by an increase in both the 0-12 and 25-36 hour prodromal time windows. Allowing a random slope, however, did not significantly add variance for these two features. For effort spent and stressors encountered, none of the contrasts significantly explained variance.

For cognitive functioning and positive affect, a decline was found in the 0-12 and 25-36 time window, but not in the 13-24 hour window (see Figure 2). These results either suggest a true cyclic pattern or they are an artifact, for example because attacks that started in the night (reported after getting up) were relatively over or underrepresented due to non-equidistant diary keeping. It was found, however, that prodromal features of night attacks were not over or underrepresented in the 13-24 hour time window. Furthermore, repeating the analyses excluding the night attacks yielded the same cyclic pattern for cognitive functioning and positive affect.

Last, item-specific post-hoc analyses were performed per time window for all prodromal characteristics assessed, since the present results represent only 47 of the 80 potential characteristics that were covered (table available on request). These analyses - corrected for the problem of multiple comparisons - yielded no additional outcomes.

## Discussion

This study prospectively identified prodromal features in migraine relative to individual interictal baseline within six consecutive 12-hour intervals prior to attack onset. Results showed that self-reported sensory sensitivity, pain/stiffness and fatigue were significantly increased, specifically within the 12 hours before attack occurrence; a tendency was found for increased negative affect in the same time window. In the same pre-attack 12-hour interval, cognitive functioning and positive affect were impaired simultaneously, but this was preceded by an earlier decline within 25-36 hours before the attack.

These findings consolidate those from prior prospective studies. According to Giffin et al. (2003) susceptibility to light and noise (sensory sensitivity), stiffness of the neck (pain/stiffness), fatigue and cognitive slowing predicted attack occurrence [7]. Wöber et al. (2007) also identified muscle tension in the neck (pain/stiffness), fatigue and psychic tension (negative affect) as attack precursors [22]. Our study added clarity regarding the predominance of these features within the last 12 hours of the migraine prodrome.

A relevant issue, also raised by Schoonman et al. (2006) [12], is whether sensory sensitivity and pain/stiffness in the hours before an attack should be considered as distinct precipitating symptoms. Photo- and phonophobia are prominent accompaniments of the attack [1], and pulsating, unilateral pain that typifies migraine may radiate to co-occurring stiffness and a-specific head pain. Future EMA studies can clarify to what extend core symptoms of the attack may appear before onset in weaker or partial expressions.

Effort spent and stressors encountered both did not predict attack occurrence, and for negative affect only a tendency was found explaining little variance. These results confirm findings of Hashizume et al. (2008) [23] that stressors encountered and negative mood were not significantly elevated in the prodromal phase. This is in harmony with the finding of Schoonman et al. (2007) who did not find evidence for a stressphysiological response before migraine attacks [29]. Although migraine patients often retrospectively report stress as a potential trigger in questionnaire studies [11,13,14,16,17,22,30], our results that stress is not elevated prior to attack occurrence raise the question whether this is in fact the case. In our view the following four considerations should be taken into account regarding this issue. First, we believe that we captured ‘stress’ quite well in our EMA diary, which permitted a distinction between stressful conditions encountered (unpleasant events, a conflict, problems, etc.), effort spent (working hard, feeling strained, exertion, etc.) and psychological stress responses subsumed under ‘negative affect’ (tension, dreariness, annoyance, anger, etc.). Second, it is a well-established finding that EMA studies are superior to retrospective studies regarding ‘recall bias’. Convictions and beliefs of migraine patients that stress acts as an attack precursor may amplify recall bias in retrospective studies, and this bias may be strengthened by the experience of stress as a consequence (instead of a precursor) of migraine. Third, we established evidence for the migraine prodrome relative to subject-specific interictal control ratings, while retrospective studies did not take interictal functioning into account. Our results are in accordance with the smaller study of Hashizume et al [23] based - as the present study - on prodromal deviations from interictal functioning. Interictal baseline may, however, not be normal in migraine. Fourth, according to our fixed factor results, psychological disregulation in terms of decreased positive affect and increased cognitive disfunction is the first sign of deviation from interictal state, which applied to the average participant. The significant random effect found for negative affect, however, shows that negative affect is increased on top of this in a subgroup of patients. This indicates individual differences in the degree to which psychological disregulation is involved in the last 12 hours of the migraine prodrome, and it underscores the need for future research aimed at identifying personalized markers of attack risk.

The present study captured cognitive-affective prodromal functioning more closely by including measures of positive affect. Additionally, the measure for cognitive functioning was derived from positive items as well. We regard the attention for positive functioning an asset of our study, and a change was found for both measures. Future research of the migraine prodrome should - instead of only focusing on negative mood or impaired thinking - extend the assessment to the full range of functioning and include positive aspects as well.

The results yielded striking similarities in the prodromal state of cognitive functioning and positive affect. Decreased functioning in both realms occurred in parallel and twice: in the 25-36 hour and - to a larger extent – in the 0-12 hour pre-attack time window. Changes were significant only as fixed effects, which means that both prodromal features apply across subjects without significant impact of individual differences. Also, results remained when repeating the analyses excluding attacks starting in the night, indicating a cyclic pattern of increasing cognitive-affective impairment within the migraine prodrome.

In summary, we conclude that online EMA is adequate to identify prodromal features in migraine, and this study is the first to show that the method can narrow and specify the migraine prodrome. The prodromal change in migraine - relative to interictal functioning - predominantly exists within the last 12 hours before attack onset. Sensory sensitivity, pain/stiffness, fatigue and (less pronounced) negative affect are elevated within the 12 hours before attack onset. Changes from interictal functioning were small when aggregated over subjects (fixed-factor analysis), partly due to substantial individual differences (random-factor analysis) indicating that these prodromal features do not operate uniformly in migraine. The impairment of cognitive-affective functioning starts earlier (up to 36 hours before the attack onset), is a general affliction, and exhibits a cyclic and progressive course. Stress - in terms of stressors encountered or effort spent - is not elevated relative to the interictal state.

We attend to four limitations of the current study. First, since a headache neurologist did not diagnose participants in all cases, the proper diagnosis may be an issue of dispute. However, participants were included based on an ICHD-II questionnaire and headache diagnostic diary, headache centers were substantially involved in recruitment, and we could approach headache specialists in case of diagnostic doubt (which happened incidentally and did not pertain to the cases included in this study). Second, participants underwent behavioral training in migraine self-management before the assessment period. The training focused on timely preventive actions (particularly through voluntary self-relaxation) and thus alerted participants to prodromal warning signs of impending attacks. This is likely to have reinforced the detection of symptoms in the present group. However, the potentially increased attention to symptoms pertained to the interictal state as well, and we assume that the effect of training on the results of the present study is corrected by the fact that prodromal features were established as deviations from interictal functioning. Third, a relatively short assessment period of three weeks was used, which did not span the menstrual cycle and also reduced the number of attacks and observations for each pre-attack window. Since we obtained 4.4 out of 6 pre-attack values on average, the missing values have hampered reliable determination of cross-level interactions. Last, the amount of variance explained by fixed factor analysis is relatively low (range 2.8-10.9%) for the prodromal features identified in this study. Allowing a random slope, however, added a substantial amount of variance explained. Strong aspects of the current study are the high compliance (89.5%), data analysis based on change scores relative to individual interictal baseline, the matching of pre-attack and control diary entries according to time point per day and weekend vs. week, separate calculations for the six 12-hour intervals of the supposed migraine prodrome, and the use of linear mixed model multilevel analysis.

Our results are of clinical relevance, particularly in preventive treatment. Knowledge of markers of a migraine attack in progression is a prerequisite of timely and targeted therapeutic actions to ameliorate the headache and prevent its occurrence where possible. Based on findings of the present study, migraine patients could be alerted that the recurrence and aggravation of cognitive-affective deterioration within one day after onset indicates attack risk within 12 hours, particularly when accompanied by (combinations of) increased sensory sensitivity, pain/stiffness and fatigue. This risk could be counteracted by concerted pharmacological and self-management actions.

The aggravation of the prodrome within the last 12 hours is subject to substantial individual differences. For example, it is accompanied by psychological disregulation in a subgroup of patients. These findings call for closer EMA research with supplementary adaptations, such as event sampling and high density assessments, to zoom in on the progression of symptoms in the last 12-hour stage of the migraine prodrome. The results also warrant more longitudinal EMA assessment to isolate potential patterns or profiles of prodromal diversity. Deeper and more detailed investigations of individual prodromal differences are of high clinical relevance. Such analyses could identify subgroups with particular prodromal features, which might emerge as phenotypical markers of migraine associated with, respectively, specific clinical expressions, genotypes or responses to treatment [4].
